# Charity and the Industrial Classes

**Published:** 1911-09-30

**Authors:** 


### Charity and the Industrial Classes.

The discontent of the medical profession with the way in which it is sweated and exploited under the guise of charity has long become chronic. We do not remember a more striking example of the manner in which the good nature of medical men is imposed on than that narrated by Dr. E. D. Kirby before the section of Medical Sociology at the annual meeting of the British Medical Association. As the facts concerned one of our largest and most progressive industrial cities, and bear very appositely on the problems of the immediate future, they are worthy of a brief recapitulation here.

In 1793 the Birmingham General Dispensary was founded " for the medical and surgical relief of poor inhabitants of the town and neighbourhood either at the Institution or at their home." For nearly eighty years these objects were duly carried out; but about 1870 certain bodies of working men began to subscribe for their own benefit to the Dispensary, and this example was soon followed by employers, who subscribed in their own names for their "works club." No one had the foresight or the energy to protest effectually on behalf of the medical profession, although it might surely have been anticipated what the result would be. At all events the works clubs, medical appointments to which had always been coveted as much more desirable than club work for Friendly Societies, soon ceased to employ medical men and began to use the General Dispensary instead. This practice, entirely foreign to the objects of the Dispensary and to the gratuitous nature of the medical attendance there, was much stimulated by the organisation of the Hospital Saturday Fund, which was established in Birmingham in 1873. For very soon the promoters of the Fund began to canvass the factories for a compulsory levy on the worker's weekly wages, and obtained it, usually one penny per week in the pound. As Dr. Kirby says, it is small wonder that the workpeople considered they had a right to free medical attendance at the various charities of the city, and exercised it freely. Then in 1893 representatives of the wage-earners arranged to attend the annual meeting of the Dispensary and swamped the ordinary subscribers, with the result that the Committee has ever since been dominated by this intelligent if somewhat selfish class.

The abuse which has thus developed is a very serious one. A very large number of good class artisans, who are said to be earning two or three pounds a week, pay their penny to the Dispensary and take care to exact a full pennyworth and more in benefits. As a matter of fact the Dispensary has a considerable income from the endowments of the charitable, so that each subscriber gets his benefits at less than cost price. With medical attendance free into the bargain, it is not surprising that the artisans of Birmingham perceive that in the Dispensary they possess a " soft thing," and support it in large numbers. Nor is it only the wage-earners who turn the Dispensary to advantage. The employers also use it as a means of sweating the profession, and Dr. Kirby tells us of one commercial magnate who is most indignant over Mr. Lloyd George's scheme, because his workmen at present obtain for fourpence (twopence contributed by themselves and twopence by the employer) all the advantages for which the Chancellor will charge ninepence.

There are, too, worse evils than the sweating of the medical profession which this system has fostered. For one thing the Friendly Societies, which do pay, however inadequately, for medical services, suffer severely from the competition of the Dispensary. The medical profession has no cause to love the Friendly Societies; but they are in many ways valuable institutions, even if they do treat their medical officers harshly. More important than this, the well-to-do artisan has actually elbowed out of the Dispensary the very class for whom it was intended. The widow and the orphan and the chronic invalid cannot get tickets, without which they cannot be seen. At the works they are told that the tickets there are only for the workpeople, and the wealthy and independent subscriber does not live in the neighbourhood and is, for them, ungetatable. That this is the fact Dr. Kirby himself vouches for; and it constitutes, as surely all must agree, a most deplorable and anomalous state of affairs. It shows quite clearly that in dealing with the modern labouring class, organised under capable leaders, the medical profession must stand together on behalf not merely of its own rights, but also of those of the really indigent and helpless

